# Cutaneous manifestations in D-2-hydroxyglutaric aciduria type 2 and response to enasidenib therapy

**DOI:** 10.1016/j.jdcr.2024.10.020

**Published:** 2024-11-08

**Authors:** Jennifer Roux, Gabrielle Brody, Brandie Metz, Jingyun Gao, Changrui Xiao, Richard C. Chang

**Affiliations:** a University of California Irvine School of Medicine, Irvine, California; b Department of Dermatology, Kaiser Permanente Los Angeles Medical Center, Los Angeles, California; c Department of Dermatology, Children’s Hospital of Orange County, Orange, California; d Pediatric Dermatology of Orange County, Irvine, California; e Department of Neurology, University of California Irvine, Irvine, California; f Metabolic Disorders Division, Children’s Hospital of Orange County, Orange, California

**Keywords:** D-2-hydroxyglutaric aciduria, dermatitis, enasidenib, pediatric

## Introduction

D-2-hydroxyglutaric aciduria (D2HGA) is a metabolic disorder characterized by elevated levels of D-2-hydroxyglutarate in the brain, urine, plasma, and cerebrospinal fluid leading to developmental delay, hypotonia, and seizures.^1^ There are 2 types of D2HGA, each resulting from mutations in different genes. D2HGA type 2 (D2HGA2) is an autosomal dominant condition caused by gain-of-function in isocitrate dehydrogenase 2 (IDH2).^2^ This gain-of-function variant has been previously reported and is known to be pathogenic. IDH2 is located in the mitochondria and converts isocitrate to alpha-ketoglutarate in the Krebs cycle. The gain-of-function variant in *IDH2* leads to the overproduction and accumulation of D-2-hydroxyglutarate from alpha-ketoglutarate, resulting in cell damage and death.^3^ Clinical manifestations of D-2-hydroxyglutarate accumulation typically present by age 2, although in less severe cases, they may emerge between ages 2 and 6.^3^

D2HGA2 is exceptionally rare, with approximately 80 reported cases worldwide.^1^ There are few reported dermatologic findings in this disease, including case reports of angiokeratomas and hemangiomas in patients with known D2HGA2.^2^^,^^4^ Here, we review 2 cases of D2HGA2 wherein the patients exhibited skin findings which showed dramatic response to IDH2 inhibitor systemic therapy.

## Cases

### Case 1

**Fig 1:**
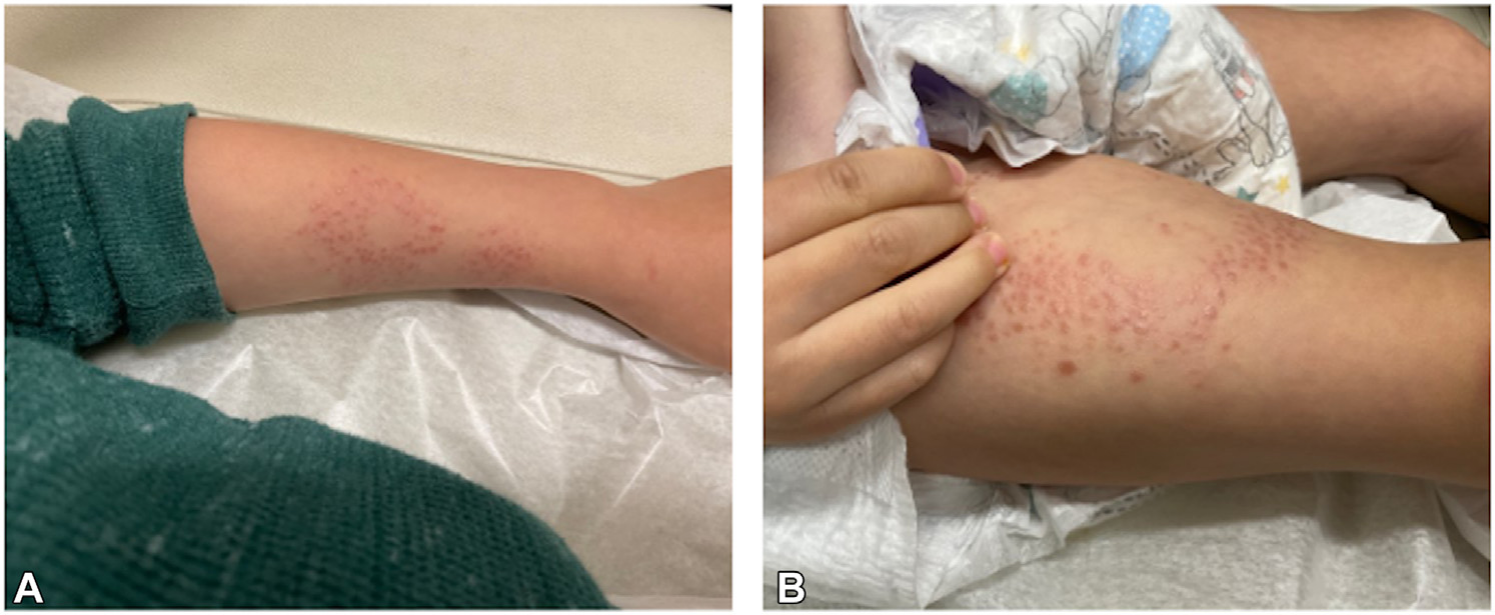
Three-year-old female with a rash characterized by confluent violaceous papules on the upper extremity (**A**) and lower extremity (**B**).

**Fig 2:**
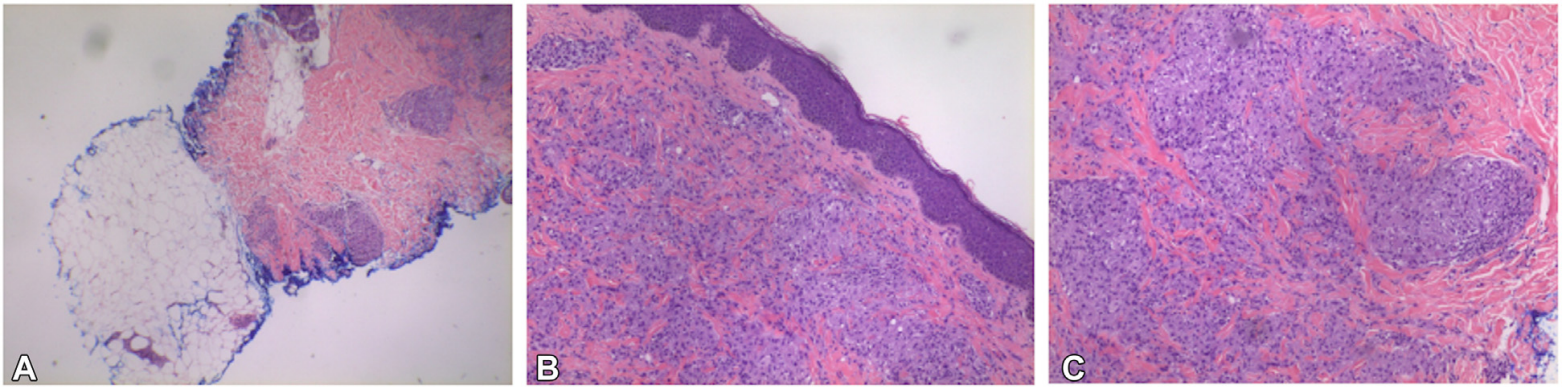
Hematoxylin and eosin, low (**A**), medium (**B**), and high (**C**) power. Noncaseating granulomas and a rare Schaumman body were seen.

A 3-year-old child presented with cerebral ventriculomegaly, hypotonia, and developmental delay. Genetic testing found a heterozygous gain-of-function variant in *IDH2 (c.419 G > A, p.R140Q)*, confirming D2HGA2. Further investigation revealed elevated urine D-2-hydroxyglutarate, diffuse supratentorial leukodystrophy, dilated cardiomyopathy, and a rash. The rash was characterized by confluent violaceous papules on the face and extremities and had been present for 1 year (Fig 1). A punch biopsy demonstrated noncaseating granulomas of the sarcoidal type with negative stains for microorganisms (Fig 2). The rash demonstrated no improvement with topical steroids (clobetasol and mometasone). Subsequent treatment with enasidenib for D2HGA2 resulted in reduced urine D-2-hydroxyglutarate and complete clearance of the rash.

### Case 2

**Fig 3:**
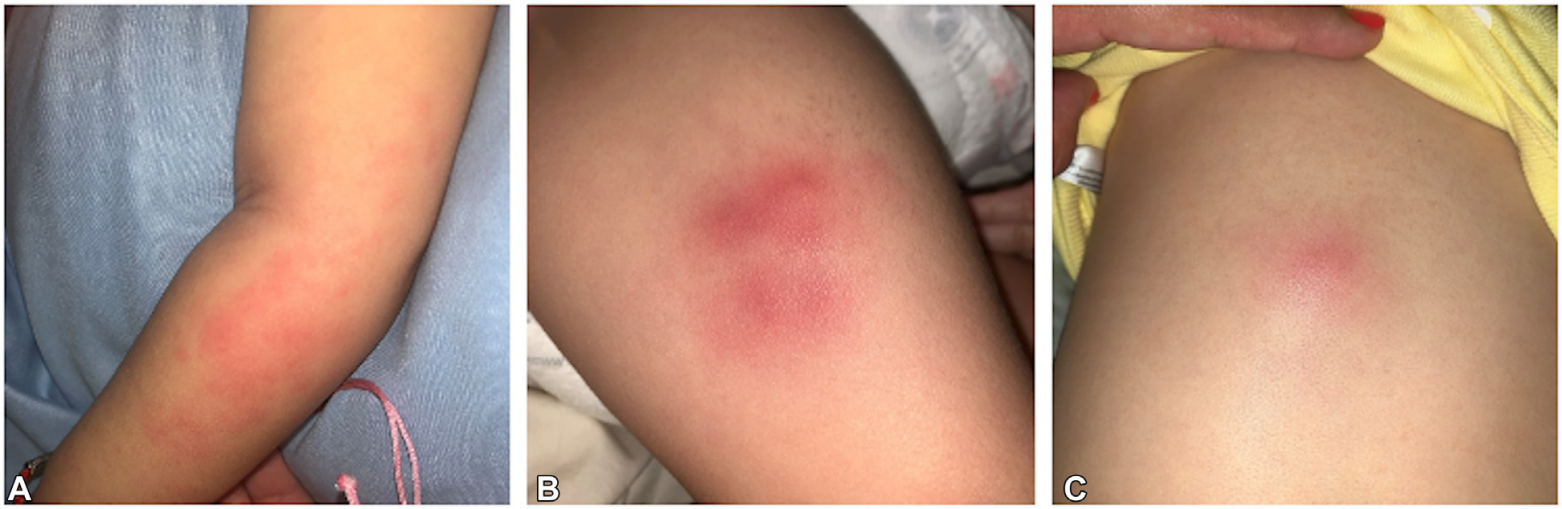
Eight-month-old infant with a recurrent rash characterized by erythematous nodules on the upper extremity (**A**), lower extremity (**B**), and trunk (**C**).

An 8-month-old infant with ventriculomegaly and developmental delay was admitted to the hospital due to infantile spasms. Laboratory tests revealed elevated D-2-hydroxyglutaric acid in the urine, and genetic analysis identified the gain-of-function variant *(c.419 G > A, p.R140Q)* in the *IDH2* gene, confirming the diagnosis of D2HGA2. Over the next year, the patient experienced recurrent episodes of erythematous nodules (Fig 3), often accompanied by fever. Clinical examination revealed erythema measuring 5 by 2.5 cm with an underlying 1.5 cm nodule. A skin biopsy revealed atypical dermal and subcutaneous histiocytic infiltrates with positive staining for CD68, CD163, and MPO, alongside occasional plasmacytoid dendritic cells indicated by CD123 staining. A myelomonocytic process was ruled out with a complete blood count with differential. The rash persisted despite treatment with topical steroids (triamcinolone). After 4 months of enasidenib therapy for D2HGA2, the patient exhibited normal D-2 hydroxyglutarate levels and complete resolution of the rash.

## Discussion

D2HGA2 is a rare inborn error of metabolism characterized by developmental delay, hypotonia, seizures, and occasionally cardiomyopathy and facial dysmorphism.^1^^,^^2^ No cases of rashes have been reported in association with D2HGA2.

In the described cases, the same gain-of-function variant was identified in *IDH2* along with elevated levels of D-2HG. Brain cells exhibit heightened vulnerability to D-2HG, explaining the predominant neurologic manifestations observed in D2HGA2.^1^ In cardiac tissue, the abundance of mitochondria and thus accrual of D-2HG is hypothesized to play a central role in the onset of cardiomyopathy in D2HGA2.^3^

The correlation between D-2HG accumulation and dermatologic symptoms in D2HGA2 remains unclear. Mutations in the *IDH2* gene have been identified in D2HGA2 patients exhibiting angiokeratomas and hemangiomas.^2^^,^^4^ It is postulated that the accumulation of D-2HG promotes angiogenesis by upregulating vascular endothelial growth factor, contributing to the development of angiokeratomas and hemangiomas.^5^ Furthermore, somatic mutations in *IDH2* have been observed in the histiocytes of granulomatous lesion in patients with myelodysplastic/myeloproliferative neoplasms.^6^ This is of interest given that histologic analysis in these 2 cases revealed histiocytic/granulomatous processes. Recent studies have highlighted the immunomodulatory role of D-2HG through its effects on macrophage polarization, histiocyte activation, epigenetic modifications, cytokine and chemokine production, oxidative stress, and chronic inflammation.^7^ These mechanisms may collectively contribute to chronic inflammatory cutaneous conditions.

Our patients demonstrated marked improvement in their rash after starting enasidenib therapy. Enasidenib selectively inhibits mutant *IDH2* and has been found to reduce D-2HG levels in D2HGA2 individuals.^8^ The resolution of these patients’ rashes with systemic D2HGA therapy supports the theory that these rashes are a primary dermatologic manifestation of their D2HGA disease.

Here, we present the first 2 cases of D2HGA2-related rashes. While the clinical and histologic presentation was different in these 2 patients, complete resolution with systemic D2HGA2 treatment is supportive evidence that the skin findings are directly caused by the underlying disease. Although the exact cause remains unclear, recognizing the association between D2HGA2 and the granulomatous/histiocytic dermatological manifestations is crucial for timely and effective treatment. Emerging reports of dermatologic findings of D2HGA2 underscore the need for comprehensive understanding of the pathophysiology to better predict the full spectrum of D2HGA2 manifestations, facilitating accurate diagnosis and effective management strategies.

## Conflicts of interest

None disclosed.
